# Linking Cellular and Mechanical Processes in Articular Cartilage Lesion Formation: A Mathematical Model

**DOI:** 10.3389/fbioe.2016.00080

**Published:** 2016-10-31

**Authors:** Georgi I. Kapitanov, Xiayi Wang, Bruce P. Ayati, Marc J. Brouillette, James A. Martin

**Affiliations:** ^1^Department of Mathematics, The University of Iowa, Iowa City, IA, USA; ^2^Program in Applied Mathematical and Computational Sciences, The University of Iowa, Iowa City, IA, USA; ^3^Department of Orthopaedics and Rehabilitation, University of Iowa Hospitals and Clinics, Iowa City, IA, USA; ^4^Department of Biomedical Engineering, The University of Iowa, Iowa City, IA, USA

**Keywords:** articular cartilage, osteoarthritis, mathematical modeling and simulation, age-structured model, reaction-diffusion model, finite element analysis

## Abstract

Post-traumatic osteoarthritis affects almost 20% of the adult US population. An injurious impact applies a significant amount of physical stress on articular cartilage and can initiate a cascade of biochemical reactions that can lead to the development of osteoarthritis. In our effort to understand the underlying biochemical mechanisms of this debilitating disease, we have constructed a multiscale mathematical model of the process with three components: cellular, chemical, and mechanical. The cellular component describes the different chondrocyte states according to the chemicals these cells release. The chemical component models the change in concentrations of those chemicals. The mechanical component contains a simulation of a blunt impact applied onto a cartilage explant and the resulting strains that initiate the biochemical processes. The scales are modeled through a system of partial-differential equations and solved numerically. The results of the model qualitatively capture the results of laboratory experiments of drop-tower impacts on cartilage explants. The model creates a framework for incorporating explicit mechanics, simulated by finite element analysis, into a theoretical biology framework. The effort is a step toward a complete virtual platform for modeling the development of post-traumatic osteoarthritis, which will be used to inform biomedical researchers on possible non-invasive strategies for mitigating the disease.

## 1 Introduction

The most common joint disease is osteoarthritis (OA), which causes joint pain and disability in those affected. It represents a growing cost to the health-care system as the incidence is expected to increase from roughly 48 million people in 2005 to 65 million by 2030 (Hootman and Helmick, 2006). A subset of these cases develop after a known trauma and are then labeled post-traumatic OA (PTOA), and despite being heavily researched, the treatment options to prevent the occurrence of OA after an injury remain limited (Anderson et al., 2011a).

This is partly because the response of articular cartilage to compressive stress is complex. Moderate physiological stresses are known to be beneficial, causing the cells in cartilage, chondrocytes, to increase production of cartilage matrix molecules (Sah et al., 1989; Quinn et al., 1998; Tomiyama et al., 2007). Severe applications of stress, such as a blunt impact injury or an intra-articular fracture, cause chondrocyte death and eventual cartilage deterioration leading to PTOA development (Anderson et al., 2011b). However, the direct cell death from these impact injuries seems to be minor, as the majority of the cell death happens hours to days after the injury (Martin et al., 2009; Goodwin et al., 2010; Tochigi et al., 2011, 2013). A complex interplay between reactive oxygen species (ROS), pro-inflammatory cytokines (PIC), and erythropoietin (EPO) seems to dominate if/how this cell death occurs and spreads (Martin et al., 2009; Ding et al., 2010; Goodwin et al., 2010; Graham et al., 2012; Wang et al., 2015). Creating a model that describes this pathway and determines thresholds that can cause the cell death to spread would provide an invaluable tool for identifying injuries that are at risk for PTOA lesion development or extrapolate treatment effects at varying doses or time scales that might not be feasible experimentally.

To that end, in the current article, we present a multiscale mathematical model of the mechanotransductive processes that result from a blunt impact with a metal indenter onto a cylindrical cartilage explant. The model consists of three scales: mechanical (tissue-level), cellular, and chemical. The mechanical component, external to the core biomathematical model and simulated with the finite element solver Abaqus™, estimates the strains resulting from the blunt impact on the cartilage explant. The cellular component categorizes the behavior and states of chondrocytes under different chemical signals. The chemical component describes these chemical signals, as well as the cytokines the chondrocytes release.

Previous attempts to model the properties of cartilage only considered its mechanical behavior (Mow et al., 1980, 1989; Lai et al., 1991). We aim to build on the biomechanical approach by adding the interplay between the chondrocytes and cytokines, resulting in articular cartilage lesions. In previous work on this topic, the model in Wang et al. (2014) considered constant cyclic loading on the cartilage, which has a different effect than the singular impact that we are concerned with in the current study. In Graham et al. (2012) and Wang et al. (2014), the authors used delay differential equations to describe the time delay in the switch of chondrocytes from one state to another. In Wang et al. (2015), the authors present an age-structured model that allows that switch to happen after a chondrocyte has been in a certain state for a certain amount of time. Age in this context is not actual cell age but how long a cell has been in its current state. This approach has led to faster computation times and greater model flexibility, and as such is used in the model in this paper. What is fundamentally different in the current model is that, unlike Wang et al. (2015), we integrate explicit mechanics into a biomathematical model in order to simulate the strain response of the tissue under singular impact. This mechanical component uses explicit finite element analysis by computing the propagation of strain through the tissue from the applied pressure and calculates the starting density distribution of necrotic cells, which initiate the cellular and chemical components of the model.

There is evidence that impact energy and the resulting mechanical stress that accompany an intraarticular fracture are a predictor of PTOA severity (Anderson et al., 2011b). Such energy has been estimated using CT scans of the fractured joint *via* finite element models (Anderson et al., 2011b). The inclusion of mechanics, and particularly stress and strain distributions, is a step to applying the modeling framework presented here to patient-dependent prediction of PTOA progression and, eventually, treatment strategy. Therefore, it is important that the presented model be viewed as an intermediate step to a comprehensive platform for biomedical research, which will require years of calibration and validation until it is applicable to patient data. Furthermore, patient and experimental data are often collected in three spatial dimensions, which necessitated the addition of spatial depth to the model.

This paper is organized as follows: Section 2 introduces the model’s equations and the numerical methods used for their solution. Section 3 describes the numerical results, and Section 4 is Discussion.

## 2 Materials and Methods

This section describes the mathematical model and the implemented numerical methods.

### 2.1 Mathematical Model

The experiment we are modeling involves dropping a metal indenter onto a cartilage explant from a drop tower. A detailed description of the experiment can be found in Wang et al. (2015). The cartilage explant is modeled as a cylinder, with the assumption of circular symmetry. This assumption reduces the model to two dimensions in space: radial (*r*) and axial (*z*, representing the depth of the cylinder). The independent variables of the system are radius (*r*), depth (*z*), time (*t*), and cell-state age (*a*). Some cells stay in a certain state only for a certain amount of time before they switch to another. This feature is modeled using age-structure, hence the use of *a*.

#### 2.1.1 Components of the Model

**Figure 1:**
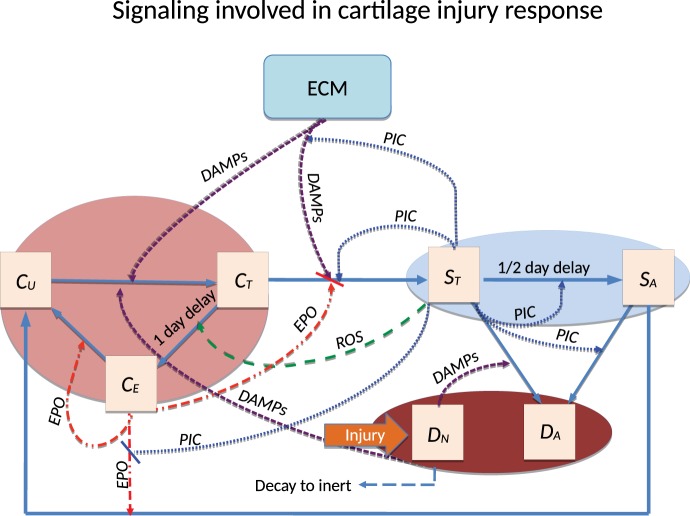
**Schematic of the articular cartilage lesion formation process due to blunt impact**. The initial injury causes cell death. As a result the necrotic cells (*D_N_*) release DAMPs, which initiate the chemical cascade leading to OA. Figure adapted from Wang et al. (2015). Table 1 contains a short description of the cell types and chemicals seen in the flowchart.

A schematic of the system is presented in Figure 1. The blunt impact causes necrosis of the chondrocytes. Necrotic chondrocytes release damage-associated molecular patterns (DAMPs), which cause the chondrocytes to release pro-inflammatory cytokines (PIC), such as tumor necrosis factor α (TNF-α) and interleukin 6 (IL-6). Inflammation leads to cell apoptosis and the degradation of the extracellular matrix (ECM). However, it also signals the release of reactive oxygen species (ROS). ROS are precursors for the release of erythropoietin (EPO), which is an anti-inflammatory cytokine that counteracts the effects of inflammation. PIC can signal cells to express erythropoietin receptor (EPOR), which makes them transit back into healthy chondrocytes if enough EPO is present. This complex feedback cycle requires two main components in our system of equations, cellular and chemical. The elements of the cellular component are:

- *C_U_*(*r*, *z*, *t*): population density (cells per unit area) of unsignaled healthy cells at a given time and location.
- *C_T_*(*r, z, a, t*): population density of healthy cells signaled by DAMPs and in the process of becoming catabolic cells. In the presence of ROS they may be signaled to release EPO (by moving to the *C_E_* class) in 20–24 h.
- *C_E_*(*r, z, t*): population density of healthy cells signaled by ROS and starting to produce EPO.
- *S_T_*(*r, z, a, t*): population density of cells in the catabolic state. Healthy cells signaled by alarmins (DAMPs) and PIC enter into the catabolic state. Catabolic cells synthesize cytokines associated with inflammation, and also produce ROS. *S_T_* cells can be signaled by the PIC to become EPOR-active cells (*S_A_*). There is a 8- to 12-h time gap before a cell expresses the EPO receptor after being signaled to become EPOR-active.
- *S_A_*(*r, z, t*): population density of EPOR-active cells. EPOR-active cells express a receptor for EPO. Catabolic cells signaled by PIC enter the EPOR-active state and may switch back to healthy state *C_U_* when signaled by EPO.
- *D_A_*(*r, z, t*): population density of apoptotic cells. Catabolic cells are signaled by DAMPs and PIC to enter the apoptotic state. EPOR-active cells will also turn to apoptosis after signaled by PIC. Apoptotic cells are omitted in the system.
- *D_N_*(*r, z, t*): population density of necrotic cells. In this model, necrotic cells emerge only due to the initial load. They release alarmins (DAMPs) into the system.

Chondrocytes exhibit minimal motility inside the ECM, so the cellular equations do not feature diffusion terms or other motility.

The elements of the chemical component are:

- *R*(*r, z, t*): concentration of reactive oxygen species (ROS). In our model, ROS signal the pre-catabolic *C_T_* cells to start releasing EPO (after becoming *C_E_* cells).
- *M*(*r, z, t*): concentration of alarmins (DAMPs) released by necrotic cells and ECM degradation. DAMPs signal healthy cells to enter a catabolic state, and together with pro-inflammatory cytokines, cause the catabolic cells *S_T_* to become apoptotic.
- *F*(*r, z, t*): concentration of general pro-inflammatory cytokines (PIC), e.g., TNF-*α* and IL-6, produced by catabolic cells (*S_T_*). They have the following effects on the system:
  – signal healthy cells (*C_T_*) to enter the catabolic state (*S_T_*),
  – signal catabolic cells (*S_T_*) to enter the EPOR-active state,
  – cause both catabolic and EPOR-active cells to become apoptotic,
  – degrade the ECM, which in turn increases the level of DAMPs, resulting in further damage to the cartilage,
  – limit the production of EPO.
- *P*(*r, z, t*): concentration of erythropoietin (EPO), exclusively produced by *C_E_* cells in this model. Inflammation can suppresses this process. EPO signals EPOR-active cells (*S_A_*) to switch back to the healthy state *C_U_*. The effects of EPO depend on its concentration.

When the concentration of EPO passes the threshold *P_c_* (Brines and Cerami, 2008), the spread of inflammation can be slowed by terminating the effect of the pro-inflammatory cytokines and DAMPs on the system. We also assume that *C_E_* cells revert to the *C_U_* state when the EPO level exceeds the *P_c_* threshold. We assume that the chemicals diffuse through the entire region. The pro-inflammatory cytokines (PIC), such as TNF-*α* and IL-6, are the main promoter of cartilage lesion formation in this model, while EPO promotes cell recovery and limits the inflammation (Eckardt et al., 1989; Brines and Cerami, 2008; Wojdasiewicz et al., 2014). The balance between these pro-inflammatory and anti-inflammatory cytokines is essential for understanding the underlying causes of OA and is an important feature of the model.

- *U*(*r, z, t*): density of the extracellular matrix (ECM). ECM is degraded by pro-inflammatory cytokines and releases DAMPs. The degradation of ECM is measured by the decrease in the concentration of SO_4_ (Rarndale et al., 1982).

ECM degradation by proteases is simplified here to be related solely to pro-inflammatory cytokines and expressed in terms of decreased sulfate (SO_4_) concentration. In cartilage, the proteoglycan groups, which comprise the majority of the ECM, contain sulfate groups and the measurement of sulfate concentration is related to ECM integrity or loss thereof. The average concentration of SO_4_ in normal undamaged cartilage is 30 g/L (Rarndale et al., 1982), which is the initial weight of ECM in this model, as seen in equation (2b). Sufficiently high EPO concentration can also block ECM degradation.

#### 2.1.2 Equations

The equations for the chemical concentrations are

(1a) $$\begin{array}{l} \partial_{t}\underset{\text{ROS}}{\underset{︸}{R\left(r,z,t\right)}}=\underset{\text{diffusion}}{\underset{︸}{\frac{1}{r}\partial_{r}\left(rK_{R}R{\left(r,z,t\right)}_{r}\right)+\partial_{\mathrm{zz}}K_{R}R\left(r,z,t\right)}}-\underset{\text{natural decay}}{\underset{︸}{\delta_{R}R\left(r,z,t\right)}}+\underset{\text{production by }S_{T}}{\underset{︸}{\sigma_{R}S_{T}\left(r,z,a,t\right)}}, \end{array}$$

(1b) $$\begin{array}{ll} \partial_{t}\underset{\text{DAMPs}}{\underset{︸}{M\left(r,z,t\right)}}=\underset{\text{diffusion}}{\underset{︸}{\frac{1}{r}\partial_{r}\left(rK_{M}M{\left(r,z,t\right)}_{r}\right)+\partial_{\mathrm{zz}}K_{M}M\left(r,z,t\right)}}-\underset{\text{natural decay}}{\underset{︸}{\delta_{M}M\left(r,z,t\right)}}+\underset{\text{production by }D_{N}}{\underset{︸}{\sigma_{M}D_{N}\left(r,z,t\right)}} & \\ \;+\underset{\text{production by ECM}}{\underset{︸}{\sigma_{U}U\left(r,z,t\right)\frac{F\left(r,z,t\right)}{\lambda_{F}+F\left(r,z,t\right)}}}, \end{array}$$

(1c) $$\begin{array}{l} \partial_{t}\underset{\text{PIC}}{\underset{︸}{F\left(r,z,t\right)}}=\underset{\text{diffusion}}{\underset{︸}{\frac{1}{r}\partial_{r}\left(rK_{F}F{\left(r,z,t\right)}_{r}\right)+\partial_{\mathrm{zz}}K_{F}F\left(r,z,t\right)}}-\underset{\text{natural decay}}{\underset{︸}{\delta_{F}F\left(r,z,t\right)}}+\underset{\text{production by }S_{T}}{\underset{︸}{\sigma_{F}S_{T}\left(r,z,a,t\right)}}, \end{array}$$

(1d) $$\begin{array}{l} \partial_{t}\underset{\text{EPO}}{\underset{︸}{P\left(r,z,t\right)}}=\underset{\text{diffusion}}{\underset{︸}{\frac{1}{r}\partial_{r}\left(rK_{P}P{\left(r,z,t\right)}_{r}\right)+\partial_{\mathrm{zz}}K_{P}P\left(r,z,t\right)}}-\underset{\text{natural decay}}{\underset{︸}{\delta_{P}P\left(r,z,t\right)}}\\ \;+\underset{\text{production by }C_{E}\text{ controlled by PIC}}{\underset{︸}{\sigma_{P}C_{E}\left(r,z,t\right)\frac{R\left(r,z,t\right)}{\lambda_{R}+R\left(r,z,t\right)}\frac{\Lambda }{\Lambda +F\left(r,z,t\right)}}}, \end{array}$$

with no flux boundary conditions on the spatial domain 0 ≤ *r* ≤ *r_max_* and 0 ≤ *z* ≤ *z_max_*. *R*(*r, z, t*)*_r_*, *M*(*r, z, t*)*_r_*, *F*(*r, z, t*)*_r_*, *P*(*r, z, t*)*_r_* are the partial derivatives of the respective variables with respect to the dimension *r*. The initial conditions are

(1e) R(r,z,0)=M(r,z,0)=F(r,z,0)=P(r,z,0)=0.

The Heaviside function used in several equations below is defined as

$$\begin{array}{l} H\left(\theta \right)=\left\{\begin{array}{cc} 1,\; & \theta \geq 0,\\ 0,\; & \theta <0. \end{array}\right. \end{array}$$

We use the Heaviside function to represent the cessation of inflammation when EPO exceeds a critical threshold (*P* > *P_c_*).

The equation for the ECM concentration is

(2a) $$\begin{array}{l} \partial_{t}\underset{\mathrm{ECM}}{\underset{︸}{U\left(r,z,t\right)}}=\underset{\text{degradation by PIC under the control of EPO}}{\underset{︸}{-\delta_{U}U\left(r,z,t\right)\frac{F\left(r,z,t\right)}{\lambda_{F}+F\left(r,z,t\right)}H\left(P_{c}-P\left(r,z,t\right)\right)}}, \end{array}$$

with initial condition

(2b) $$U\left(r,z,0\right)=30\text{mg}∕\text{c}{\text{m}}^{3}.$$

The equations for the healthy cell population densities are

(3a) $$\begin{array}{l} \partial_{t}C_{U}\left(r,z,t\right)=\underset{S_{A}\overset{\text{EPO}}{\underset{\;}{\to }}C_{U}}{\underset{︸}{\alpha_{1}S_{A}\left(r,z,t\right)\frac{P\left(r,z,t\right)}{\lambda_{P}+P\left(r,z,t\right)}}}+\underset{C_{E}\overset{\text{EPO}}{\underset{\;}{\to }}C_{U}}{\underset{︸}{\alpha_{2}H\left(P\left(r,z,t\right)-P_{c}\right)C_{E}\left(r,z,t\right)}}-\underset{C_{U}\overset{\text{DAMPs}}{\underset{\;}{\to }}C_{T}}{\underset{︸}{\beta_{13}C_{U}\left(r,z,t\right)\frac{M\left(r,z,t\right)}{\lambda_{M}+M\left(r,z,t\right)}}}, \end{array}$$

(3b) $$\begin{array}{llll} & \partial_{t}C_{T}\left(r,z,a,t\right)+\partial_{a}C_{T}\left(r,z,a,t\right)=-\underset{C_{T}\overset{\text{DAMPs}}{\underset{\;}{\to }}S_{T}}{\underset{︸}{\beta_{11}\frac{M\left(r,z,t\right)}{\lambda_{M}+M\left(r,z,t\right)}H\left(P_{c}-P\left(r,z,t\right)\right)C_{T}\left(r,z,a,t\right)}}\; & & \;\\ & -\underset{C_{T}\overset{\text{PIC}}{\underset{\;}{\to }}S_{T}}{\underset{︸}{\beta_{12}\frac{F\left(r,z,t\right)}{\lambda_{F}+F\left(r,z,t\right)}H\left(P_{c}-P\left(r,z,t\right)\right)C_{T}\left(r,z,a,t\right)}}-\underset{C_{T}\overset{\text{ROS}}{\underset{\;}{\to }}C_{E}}{\underset{︸}{\kappa_{1}\gamma \left(a-\tau_{2}\right)\frac{R\left(r,z,t\right)}{\lambda_{R}+R\left(r,z,t\right)}C_{T}\left(r,z,a,t\right)}}, \end{array}$$

(3c) $$\begin{array}{l} \partial_{t}C_{E}\left(r,z,t\right)=\underset{C_{T}\overset{\tau_{2}\text{ delay}}{\underset{\;}{\to }}C_{E}}{\underset{︸}{\int_{0}^{\infty }\kappa_{1}\gamma \left(a-\tau_{2}\right)\frac{R\left(r,z,t\right)}{\lambda_{R}+R\left(r,z,t\right)}C_{T}\left(r,z,a,t\right)\mathrm{da}}}\\ \;-\underset{C_{E}\overset{\text{EPO}}{\underset{\;}{\to }}C_{U}}{\underset{︸}{\alpha_{2}H\left(P\left(r,z,t\right)-P_{c}\right)C_{E}\left(r,z,t\right)}}. \end{array}$$

The function γ(*a*) above represents the sharp age-dependent transition of cells from one state to another [in equation (3b) the transition from *C_T_* to *C_E_* and later, in equation (4a), the transition from *S_T_* to *S_A_*], in order to model the delay between the signal and the state-switch. It is given by:

$$\gamma \left(a-a_{\text{max}}\right)=\frac{\gamma_{0}}{\sigma }\left(\tanh\left(\frac{a-a_{\text{max}}}{\sigma }\right)+1\right),$$

where *a*_max_ is the state-age at which the cells switches states, γ_0_ gives the height and *σ* gives the spread of the function. The form of the function is taken from Wang et al. (2015) and the γ_0_ and *σ* values are given in Table 2.

The single blunt impact is modeled using finite element analysis to simulate the impact of the laboratory experiment’s indenter onto the simulated cartilage explant. The tissue strains resulting from the impact are calculated, and from them we calculate, using the function Γ below, the resulting fraction of necrotic (dead) cells, *D_N_*, due to the impact. This fraction of dead cells (the remaining cells are considered normal, or *C_U_*) determines the initial conditions of the system [equations (3d) and (5b)], and does not play any further part in the model since there is no subsequent loading.

$$\Gamma \left(\epsilon ,r,z\right)=0.01p_{0}\left(e^{K_{U}\epsilon }-e^{10K_{U}}\right),$$

where *ϵ* is the absolute value of the position dependent axial (vertical) strain resulting from the deformation of the cartilage from the initial load, in %. The constants *P*_0_ and *K_U_* are parameter fitting constants. The form of the function is taken from Brouillette et al. (2013) with the assumption that the strain is higher than 10%. Otherwise we assume no cell death due to strain. The value of 10% is arbitrary but moderate strains (10–15%) seem to be beneficial to cartilage biosynthesis and do not seem to be associated with cell death (Martin and Buckwalter, 2012; Brouillette et al., 2013; Sanchez-Adams et al., 2014). Therefore, the initial fraction of healthy cells is 1 – fraction of necrotic cells. Our assumption is that the initial cell density is 100,000 cells/cm^3^. This leads to the initial condition for healthy *C_U_* cells:

(3d) $$C_{U}\left(r,z,0\right)=\left(1-\Gamma \left(\epsilon ,r,z\right)\right)\left(100,000\;\text{cells}∕\text{c}{\text{m}}^{3}\right).$$

The rest of the cells are assumed necrotic (dead), which is expressed in the initial condition for *D_N_* cells in equation (5b). The age-of-state *a* boundary condition is

(3e) $$\begin{array}{l} C_{T}\left(r,z,0,t\right)=\underset{C_{U}\overset{\text{DAMPs}}{\underset{\;}{\to }}C_{T}}{\underset{︸}{\beta_{13}C_{U}\left(r,z,t\right)\frac{M\left(r,z,t\right)}{\lambda_{M}+M\left(r,z,t\right)}}}, \end{array}$$

and the initial condition is

(3f) $$C_{T}\left(r,z,a,0\right)=C_{E}\left(r,z,0\right)=0.$$

The equations for the sick cell population densities are

(4a) $$\begin{array}{llll} & \partial_{t}S_{T}\left(r,z,a,t\right)+\partial_{a}S_{T}\left(r,z,a,t\right)=-\underset{S_{T}\overset{\text{PIC, DAMPs}}{\underset{\;}{\to }}D_{A}}{\underset{︸}{\mu_{\mathrm{ST}}\frac{F\left(r,z,t\right)}{\lambda_{F}+F\left(r,z,t\right)}\frac{M\left(r,z,t\right)}{\lambda_{M}+M\left(r,z,t\right)}S_{T}\left(r,z,a,t\right)}}\; & & \\ & \;-\underset{S_{T}\overset{\text{PIC}}{\underset{\;}{\to }}S_{A}}{\underset{︸}{\kappa_{2}\cdot \gamma \left(a-\tau_{1}\right)\frac{F\left(r,z,t\right)}{\lambda_{F}+F\left(r,z,t\right)}S_{T}\left(r,z,a,t\right)}}, \end{array}$$

(4b) $$\begin{array}{llll} & \partial_{t}S_{A}\left(r,z,t\right)=\underset{S_{T}\overset{\tau_{1}\text{ delay}}{\underset{\;}{\to }}S_{A}}{\underset{︸}{\int_{0}^{\infty }\;\kappa_{2}\;\cdot \;\gamma \left(a\;-\;\tau_{1}\right)\frac{F\left(r,z,t\right)}{\lambda_{F}+F\left(r,z,t\right)}H\left(P_{c}\;-\;P\left(r,z,t\right)\right)S_{T}\left(r,z,a,t\right)\mathrm{da}}}\; & & \;\\ & -\underset{S_{A}\overset{\text{EPO}}{\underset{\;}{\to }}C_{U}}{\underset{︸}{\alpha_{1}S_{A}\left(r,z,t\right)\frac{P\left(r,z,t\right)}{\lambda_{P}+P\left(r,z,t\right)}}}-\underset{S_{A}\overset{\text{PIC}}{\underset{\;}{\to }}D_{A}}{\underset{︸}{\mu_{S_{A}}\frac{F\left(r,z,t\right)}{\lambda_{F}+F\left(r,z,t\right)}H\left(P_{c}-P\left(r,z,t\right)\right)S_{A}\left(r,z,t\right)}}, \end{array}$$

with age-of-state *a* boundary condition

(4c) $$\begin{array}{ll} & S_{T}\left(r,z,0,t\right)=\int_{0}^{\infty }(\underset{C_{T}\overset{\text{DAMPs}}{\underset{\;}{\to }}S_{T}}{\underset{︸}{\beta_{11}\frac{M\left(r,z,t\right)}{\lambda_{M}+M\left(r,z,t\right)}H\left(P_{c}-P\left(r,z,t\right)\right)}}+\underset{C_{T}\overset{\text{PIC}}{\underset{\;}{\to }}S_{T}}{\underset{︸}{\beta_{12}\frac{F\left(r,z,t\right)}{\lambda_{F}+F\left(r,z,t\right)}H\left(P_{c}-P\left(r,z,t\right)\right))}}C_{T}\left(r,z,a,t\right)\mathrm{da}, \end{array}$$

and initial condition

(4d) $$S_{T}\left(r,z,a,0\right)=S_{A}\left(r,z,0\right)=0.$$

We track necrotic cells using

(5a) $$\partial_{t}D_{N}\left(r,z,t\right)=-\underset{\text{natural}\;\text{decay}}{\underset{︸}{\mu_{D_{N}}D_{N}\left(r,z,t\right)}},$$

with initial condition

(5b) $$D_{N}\left(r,z,0\right)=\Gamma \left(\epsilon ,r,z\right)\left(100,\;000\;\text{cells}∕\text{c}{\text{m}}^{3}\right).$$

There is no equation for apoptotic cells; these are considered removed from the system.

Short descriptions of all variables are in Table 1.

**Table 1:** **Cell types and variable meaning**.

Variable	Description
*C_U_*	Healthy unsignaled chondrocytes
*C_T_*	Chondrocytes signaled by DAMPs to become catabolic
*C_E_*	Chondrocytes that produce EPO
*S_T_*	Catabolic cells that release PIC and ROS
*S_A_*	EPOR-active cells, can switch back to *C_U_* if signaled by EPO
*D_N_*	Necrotic cells. Formed by initial strain, release DAMPs
*D_A_*	Apoptotic cells, not included in the model
*R* (ROS)	Reactive oxygen species. Signal *C_E_* cells to release EPO
*M* (DAMPs)	Damage-associated molecular patterns. Alarmins released by *D_N_* and the ECM as a result of the impact
*F* (PIC)	Pro-inflammatory cytokines. Signal healthy cells to become catabolic and catabolic cells to become apoptotic. Degrade the ECM
*P* (EPO)	Erythropoietin. Anti-inflammatory cytokine. Diminishes the effects of inflammation
*U* (ECM)	Extracellular matrix. Chondrocytes live within it. Subject to degradation from the stress application and PIC

#### 2.1.3 Numerical Implementation

Our computations are done in two main stages: a finite element analysis of the strain due to the impact using the commercial software Abaqus™, followed by a simulation of the cellular and biochemical response of the explant using our own software.

Abaqus™ is a finite element analysis (FEA) software developed by Dassault Systèmes. It is generally used in a variety of engineering projects, particularly for predicting mechanical stresses and strains on automotive designs under static and dynamics loads. Because of its accuracy and wide material modeling capability, it has become one of the most widely used finite element solvers in biomedical fields.

Our in-house software features a step-doubling alternating-direction implicit (ADI) method, described in mathematical detail in Ayati and Dupont (2005) and Ayati et al. (2006), for the time and 2D space integration. The age discretization is done through a “natural-grid” Galerkin method, which to our knowledge remains the state of the art for solving partial differential equations that depend on age as well as time and space. The mathematical underpinnings and general description of the natural-grid methods are in Ayati (2000, 2007a) and Ayati and Dupont (2002, 2009). The natural-grid approach has been used for the modeling and simulation of a range of systems, such as *Proteus mirabilis* swarm colony development (Ayati, 2006, 2007b, 2009), avascular tumor invasion (Ayati et al., 2006), biofilm persistence and senescence (Ayati and Klapper, 2007; Klapper et al., 2007), bacterial dormancy (Ayati, 2012; Ayati and Klapper, 2012), and now articular cartilage lesion formation (Wang et al., 2015).

The in-house software differs from the one in Wang et al. (2015) by adding an extra dimension (depth) to the model. The ADI method had not been done in the context of OA modeling, so the whole software structured was modified to accommodate the new feature. This is also part of the reason why the sensitivity analysis we conducted does not significantly differ from previous work – we needed to verify that adding the new dimension would not necessitate parameter changes, which is hard to predict in a complex system like ours.

#### 2.1.4 Mechanical Component and Strain Simulation

The mechanical component of the modeling and simulation involves the calculations of different strains across the spatial domain of the cartilage explant and is used in the initial conditions [equations (3d) and (5b) above]. For the purposes of the current model, the explant is considered to be homogenized cartilage tissue. This is a major simplifying assumption of the model. In reality, cartilage is composed of several layers with different mechanical stiffness and a cartilage explant typically contains around 10–20% cartilage (1–2 mm), while the rest is subchondral bone. Another assumption is that the cartilage disk exhibits linearly elastic behavior. Linear elasticity is generally modeled using hyperbolic partial differential equations. Since the external pressure only affects the initial conditions, there is no feedback between our parabolic system and the strains resulting from the initial impact. Therefore, the strain at each point is constant relative to the system.

The parameters used to for the mechanical simulation were adopted from the laboratory experiment done in Wang et al. (2015). In short, a cartilage explant of size 25 mm × 25 mm × 10 mm was subjected to an impact from a drop tower indenter. The height of the tower was 50 mm and the energy of the impact was 2.13 J/cm^2^. To model the indenter’s impact on the explant, the commercial finite element solver Abaqus™ was used to simulate dynamic pressure, applied for a period of 0.001 s onto a rectangular block, and record the resulting displacements over the spatial domain, from which we then calculated the axial strains. The axial strains were calculated by computing the ratio of vertical displacement and the vertical position of the node at which the displacement was measured. The Abaqus™ simulation was done using a rectangle of dimensions 2.5 cm × 1.0 cm (the model assumes radial symmetry, while the experimental explants are rectangular prisms). A pressure of 0.436 MPa was applied onto a line of length 5.5 mm (to simulate an indenter) in the center top side of the rectangle. The value was chosen to reflect the laboratory experiment – the pressure resulting from 2.13 J/cm^2^ dropped from 5 cm is 0.436 MPa. Since our model is two-dimensional in space, a two-dimensional Abaqus™ simulation is sufficient. In order to simulate the impact of the indenter, the load is of the instantaneous pressure type. The Abaqus™ output is the axial displacement, *U*_2_, at each point of the rectangle. The mesh contains over 250,000 grid points. Because of the symmetry of the results, we only recorded the right half of the rectangle’s strains and used those data in the initial conditions of our model.

The addition of the external FE-obtained strains required a major rewrite and modification of the in-house software. The strain deck is inputted as a data file, which is searched through to find the closest strain value to the spatial point at hand. The strain values are used as input inside the functions related to initial cell distributions, and the functions that represent the differential equations of the model. None of these had to be done in Wang et al. (2015) and are new work.

#### 2.1.5 Parameter Estimation

All parameters and a reference for their value are listed in Table 2. A detailed reasoning for selecting some of the parameters based on the literature is presented in Wang et al. (2014). The strain distribution across the simulated cylinder was computed by Abaqus™ and required several parameters related to the physical properties of cartilage: Young’s modulus, Poisson ratio, and density. Since cartilage has a heterogeneous structure, these parameters can be estimated within a range. The density we used for the simulation was chosen from Mansour (2003) and, because we are modeling a blunt impact, the Young’s modulus and Poisson ratio were taken from Jin and Lewis (2004). The values of these parameters are also in Table 2.

**Table 2:** **Table of parameters**.

Parameter	Value	Units	Reason
*K_R_*	0.1	$\frac{\text{c}{\text{m}}^{\text{2}}}{\text{day}}$	Determined in Graham et al. (2012)
*K_M_*	0.05	$\frac{\text{c}{\text{m}}^{\text{2}}}{\text{day}}$	Determined in Graham et al. (2012)
*K_P_*	0.005	$\frac{\text{c}{\text{m}}^{\text{2}}}{\text{day}}$	Determined in Graham et al. (2012)
*K_F_*	0.05	$\frac{\text{c}{\text{m}}^{\text{2}}}{\text{day}}$	Determined in Graham et al. (2012)
*δ_R_*	60	$\frac{\text{1}}{\text{day}}$	Determined in Wang et al. (2014)
*δ_M_*	0.5545	$\frac{\text{1}}{\text{day}}$	Determined in Wang et al. (2014)
*δ_F_*	0.1664	$\frac{\text{1}}{\text{day}}$	Determined in Wang et al. (2014)
*δ_P_*	3.326	$\frac{\text{1}}{\text{day}}$	Determined in Wang et al. (2014)
*δ_U_*	0.0193	$\frac{\text{1}}{\text{day}}$	Determined in Wang et al. (2014)
*σ_R_*	0.0024	$\frac{\text{nanomolar}\cdot \text{c}{\text{m}}^{\text{3}}}{\text{day}\cdot \text{cells}}$	Determined in Wang et al. (2014)
*σ_M_*	5.17 × 10^−7^	$\frac{\text{nanomolar}\cdot \text{c}{\text{m}}^{\text{3}}}{\text{day}\cdot \text{cells}}$	Determined in Wang et al. (2014)
*σ_F_*	2.35 × 10^−7^	$\frac{\text{nanomolar}\cdot \text{c}{\text{m}}^{\text{3}}}{\text{day}\cdot \text{cells}}$	Determined in Wang et al. (2014)
*σ_P_*	4.2 × 10^−5^	$\frac{\text{nanomolar}\cdot \text{c}{\text{m}}^{\text{3}}}{\text{day}\cdot \text{cells}}$	Determined in Wang et al. (2014)
*σ_U_*	0.0154	$\frac{\text{nanomolar}\cdot \text{c}{\text{m}}^{3}}{\text{day}\cdot \text{cells}}$	Determined in Wang et al. (2014)
Λ	0.5	nanomolar	Estimated
*λ_R_*	5	nanomolar	Estimated
*λ_M_*	0.5	nanomolar	Estimated
*λ_F_*	0.5	nanomolar	Estimated
*λ_P_*	0.5	nanomolar	Estimated
*α*_1_	1	$\frac{\text{1}}{\text{day}}$	Estimated
*α*_2_	1	$\frac{\text{1}}{\text{day}}$	Estimated
*β*_11_	100	$\frac{\text{1}}{\text{day}}$	Estimated
*β*_12_	50	$\frac{\text{1}}{\text{day}}$	Estimated
*β*_13_	10	$\frac{\text{1}}{\text{day}}$	Estimated
*κ*_1_	10	$\frac{\text{1}}{\text{day}}$	Estimated
*κ*_2_	10	$\frac{\text{1}}{\text{day}}$	Estimated
*P_c_*	1	nanomolar	Determined in Wang et al. (2014)
*μS_T_*	0.5	$\frac{\text{1}}{\text{day}}$	Estimated
*μS_A_*	0.1	$\frac{\text{1}}{\text{day}}$	Estimated
*μD_N_*	0.05	$\frac{\text{1}}{\text{day}}$	Estimated
*τ*_1_	0.5	days	Determined in Graham et al. (2012)
*τ*_2_	1	days	Determined in Graham et al. (2012)
*γ*_0_	1		Determined in Wang et al. (2015)
*σ*	0.1		Determined in Wang et al. (2015)
*P_0_*	1		Determined in Brouillette et al. (2013)
*δ_U_*	0.0545		Determined in Brouillette et al. (2013)
Cartilage density	1600	kg/m^3^	Taken from Mansour (2003)
Cartilage Young’s modulus	1.8	MPa	Taken from Jin and Lewis (2004)
Cartilage Poisson ratio	0.5		Taken from Jin and Lewis (2004)

### 2.2 Error Analysis

To estimate relative errors, we compare the result of the default run to three runs using refined discretization: with halved Δ*a*, with both Δ*r* and Δ*z* halved, and halving the time step tolerance. For each comparison, the relative errors were calculated using standard *L*_2_ norms of *C_U_*, *S_T_* (integrated over age), and EPO at the final time node (14 days) by the formula *rel err* = *||X_b_* − *X_c_*||_2_/||*X_b_*||_2_, where *X_b_* is the value of *C_U_*, *S_T_*, EPO from the default parameter run and *X_c_* is the respective value from the comparison run. The *L_2_* norms were taken over all spatial nodes. The reported relative error was the maximum of the three relative errors.

## 3 Results

### 3.1 Computational Results and Experimental Validation

**Figure 2:**
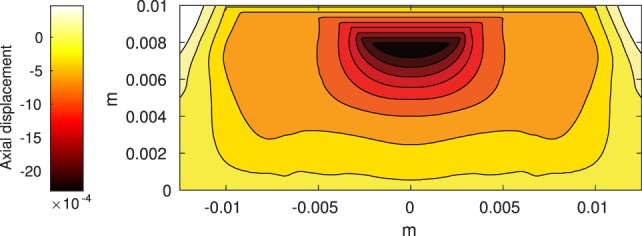
**Axial displacement on the cartilage rectangle, plotted with MATLAB, using raw data from Abaqus™**.

**Figure 3:**
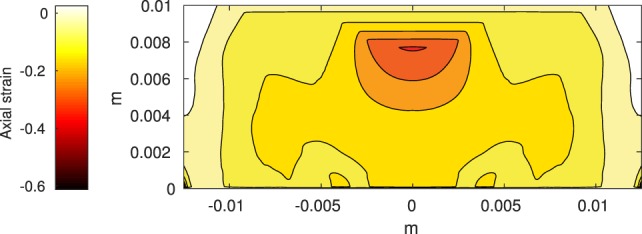
**Axial strain on the cartilage rectangle, calculated from raw displacement data from Abaqus™**.

**Figure 4:**
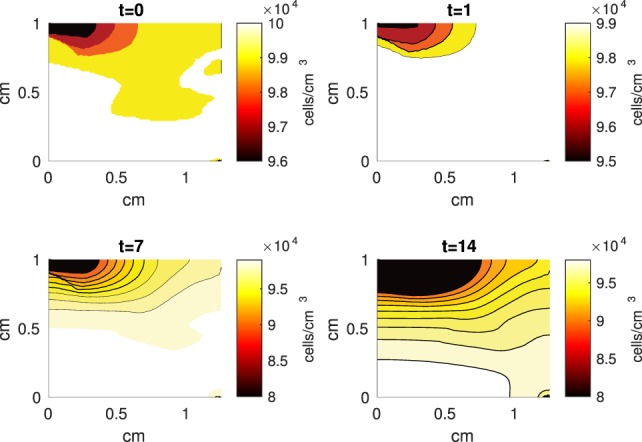
**A contour graph of the density of the normal healthy cells (*C_U_*) in the 2D model at 0, 1, 7, and 14 days**. The contour plot shows the decrease of *C_U_* cell density in the rectangular representation of the cylindrical explant. The lowest number of cells is in the upper left corner, the area of the initial contact. The units are cells/cm^3^.

**Figure 5:**
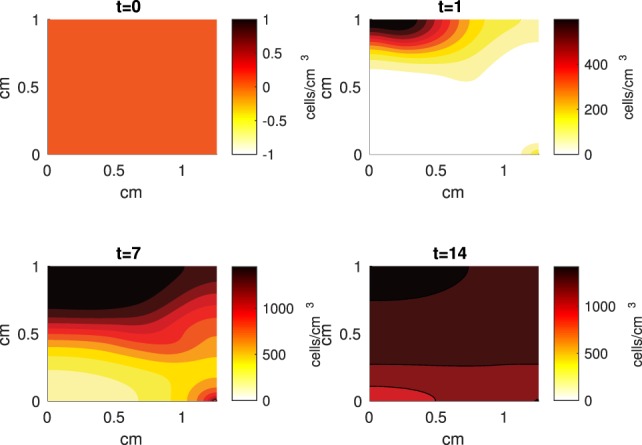
**A contour graph of the density of the healthy transitioning to catabolic cells (*C_T_*) in the 2D model at 0, 1, 7, and 14 days**. The units are cells/cm^3^.

**Figure 6:**
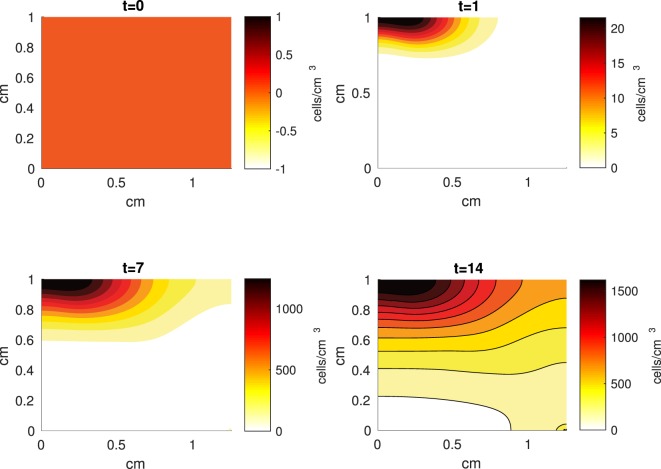
**A contour graph of the density of the catabolic cells producing pro-inflammatory cytokines (*S_T_*) in the 2D model at 0, 1, 7, and 14 days**. The units are cells/cm^3^.

**Figure 7:**
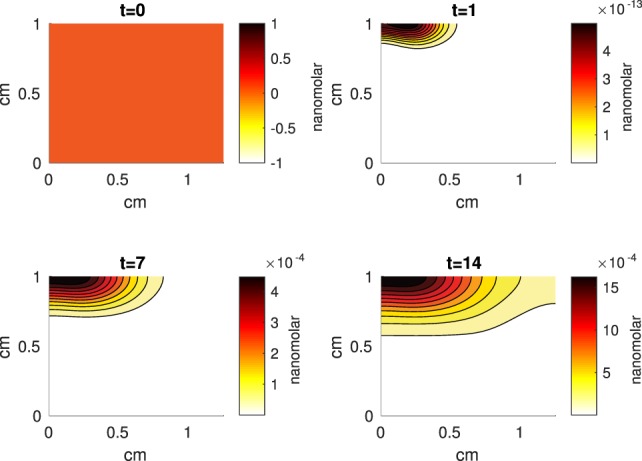
**A contour graph of the concentration of erythropoietin (EPO) in the 2D model at 0, 1, 7, and 14 days**. The units are nanomolar.

**Figure 8:**
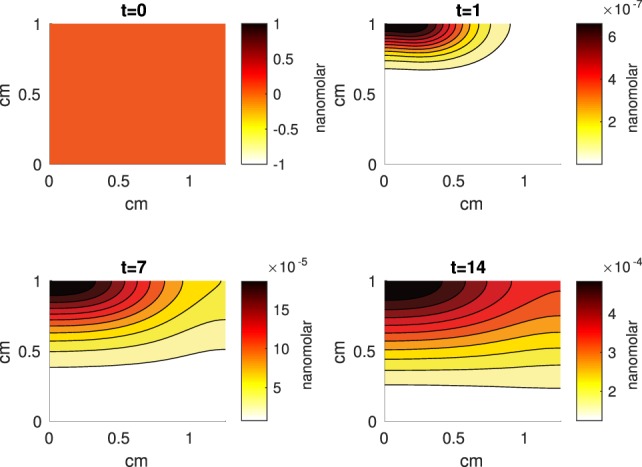
**A contour graph of the concentration of pro-inflammatory cytokines (PIC) in the 2D model at 0, 1, 7, and 14 days**. The units are nanomolar.

**Figure 9:**
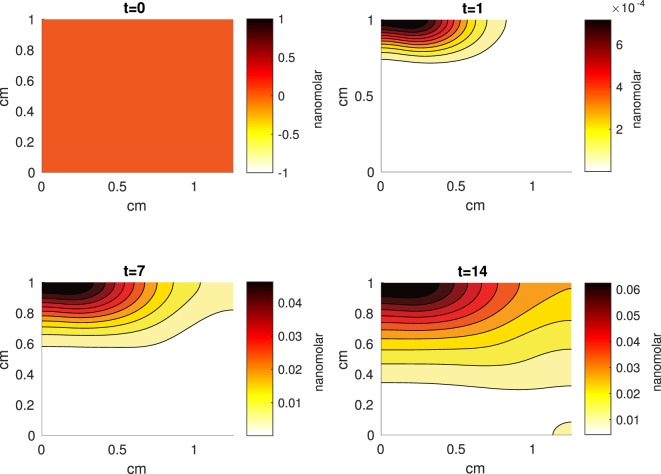
**A contour graph of the concentration of reactive oxygen species (ROS) in the 2D model at 0, 1, 7, and 14 days**. The units are nanomolar.

**Figure 10:**
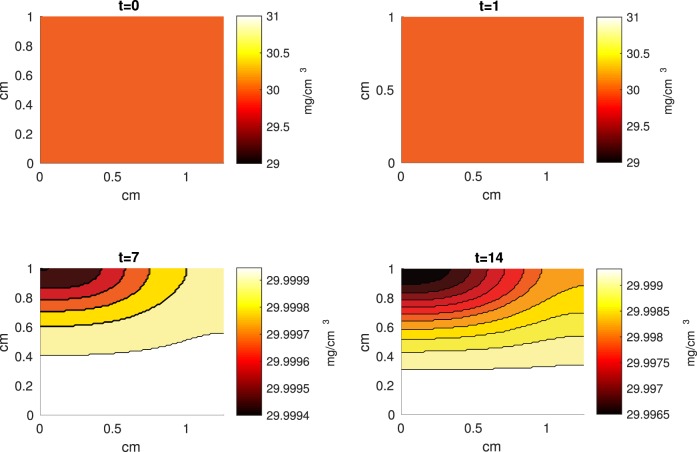
**A contour graph of the extracellular matrix (ECM) density in the 2D model at 0, 1, 7, and 14 days**. The units are mg/cm^3^.

The Abaqus™ displacement output can be seen in Figure 2. The calculated strains are in Figure 3. These strains were used as input into the initial conditions [equations (3d) and (5b)]. The results of the simulations with the default parameters are presented in Figures 4–10. Overall, according to the model, an impact of 0.436 MPa does not seem to cause significant damage to the ECM during the first 14 days, as indicated by Figure 10. However, the pressure is still sufficient to trigger the cascade of chemical processes that can lead to the development of OA. In particular, there are small amounts of PIC (evident in Figure 8) and ROS (Figure 9) released, and there is a transition of *C_U_* cells into pre-catabolic (*C_T_*) and eventually catabolic (*S_T_*) cells (Figures 5 and 6). The chemical and cellular behavior as a whole is characterized by higher densities (besides *C_U_*) and concentrations near the area of contact and diffusion along the cartilage radius. There is a biochemical response to the impact, but in this particular case, it does not cause disease, at least as indicated by the relatively undamaged ECM at the 14-day mark. Further figures from our results, particularly average densities along the radius of the explant, can be found in Supplementary Material.

### 3.2 Error Results

The maximal relative error for the half Δ*a* was 0.000055. The maximal relative error for the halved Δ*r* and Δ*z* was 0.03 and the maximal relative error for the halved time interval tolerance was 0.00016. All of these maximal errors were calculated from the *S_T_* cell class.

### 3.3 Sensitivity Analysis

While some of the variables were derived from the experimental literature, most were estimated from previous computational work. Table 3 presents the parameters and their respective default values and the values within the range over which they caused notable changes in the model’s results. When varying a parameter, we set all other parameters at their default values. Table 3 also presents cell population and chemical concentration results from increasing/decreasing the set parameters, according to relative comparison to the default case. *C_U_* and *D_N_* densities are omitted. Most of the parameter perturbations did not lead to vast qualitative changes in the behavior of the system; the changes did not generally alter the shape of the graphs or the relationship between the values. There were some quantitative differences between runs. Given that many of our parameters are estimated, better approximations can be a topic for future work. However, the model captures, at least qualitatively, expected chemical and cellular behaviors that can result after an injurious blunt impact. The concentration of ECM is also omitted – none of the parameter perturbations lead to a notable decrease in 14 days.

**Table 3:** **Table of parameter perturbation and its effect on numerical outcomes in cellular density and chemical concentration**.

Variable	EP	HP	MP	MN	HN
*C_T_*	*β*_13_ = 20	*β*_13_ = 15	*λ_F_* = 0.3	*β*_13_ = 5	*β*_13_ = 1
	*λ_F_* = 0.1	*κ*_1_ = 1		*λ_M_* = 0.9	*λ_M_* = 0.1
	*λ_M_* = 0.3			*λ_R_* = 1	
*C_E_*	*β*_13_ = 20	*β*_13_ = 16		*λ_M_* = 0.7	*β*_13_ = 1, 5
	*λ_F_* = 0.1				*κ*_1_ = 1
	*λ_M_* = 0.1, 0.3				*λ_M_* = 0.9
*S_T_*	*β*_13_ = 20	*β*_11_ = 150	*κ*_2_ = 1	*β*_13_ = 50	*β*_13_ = 1,5
	*κ*_1_ = 1	*β*_13_ = 15		*λ_M_* = 0.7	
	*λ_F_* = 0.1	*λ_F_* = 0.3	*μ_DN_* = 0.01	*λ_R_* = 1	*λ_M_* = 0.9
	*λ_M_* = 0.1, 0.3	
*S_A_*	*β*_13_ = 15,20	*β*_11_ = 150	*β*_11_ = 125	*β*_11_ = 75	*β*_11_ = 50
	*κ*_1_ = 1	*μ_DN_* = 0.01	*κ*_1_ = 5	*κ*_1_ = 20	*β*_13_ = 1,5
	*λ_F_* = 0.1, 0.3		*κ*_2_ = 20	*κ*_2_ = 5	*κ*_2_ = 1
	*λ_M_* = 0.1, 0.3		*λ_R_* = 9	*λ_F_* = 0.7	*λ_F_* = 0.9
				*μ_DN_* = 0.09	*λ_M_* = 0.7, 0.9
					*λ_R_* = 1
*M*	*β*_13_ = 20	*λ_F_* = 0.3	*β*_11_ = 150	*β*_11_ = 50	*β*_13_ = 1
(DAMPs)	*λ_F_* = 0.1		*β*_13_ = 15	*β*_13_ = 5	
	*λ_M_* = 0.1, 0.3		*κ*_1_ = 1,5	*λ_F_* = 0.9	*λ_M_* = 0.9
			*μ_DN_* = 0.01	*λ_M_* = 0.7	
*F* (PIC)	*β*_13_ = 20	*β*_11_ = 150	*λ_F_* = 0.3	*β*_11_ = 50	*β*_13_ = 1,5
	*λ_F_* = 0.1	*β*_13_ = 15	*μ_DN_* = 0.01	*λ_M_* = 0.7	*λ_M_* = 0.9
	*λ_M_* = 0.1, 0.3	*κ*_1_ = 1		*λ_R_* = 1	
*P* (EPO)	*β*_13_ = 15,20	*λ_F_* = 0.3	*β*_11_ = 150	*β*_11_ = 50	*β*_13_ = 1, 5
	*λ_F_* = 0.1	*κ*_2_ = 1		*λ_R_* = 9	
	*λ_M_* = 0.1, 0.3	*μ_DN_* = 0.01	*λ_R_* = 3	*μ_DN_* = 0.09	*λ_M_* = 0.7, 0.9
	*λ_R_* = 1	
*R* (ROS)	*β*_13_ = 20	*β*_11_ = 150	*κ*_2_ = 1	*β*_11_ = 50	*β*_13_ = 1,5
	*κ*_1_ = 1	*β*_13_ = 15	*μ_DN_* = 0.09	*λ_M_* = 0.7	*λ_M_* = 0.9
	*λ_F_* = 0.1	*λ_F_* = 0.3		*λ_R_* = 1	
	*λ_F_* = 0.1, 0.3				

*The relative effects the perturbations have on the variable values are divided into five categories: EP, extremely positive effect (over 100% relative increase); HP, highly positive effect (50–100% relative increase); MP, moderately positive (30–50% relative increase); MN, moderately negative (30–50% relative decrease); HN, highly negative (50–100% relative decrease)*.

Notable mentions among the parameters that produced large changes are *β*_11_, *β*_13_, *λ_F_*, and *λ_M_*. The parameter *β*_11_ affects the transition of *C_T_* cells to *S_T_* cells under the effect of DAMPs. The parameter *β*_13_ is the rate of *C_U_* cells transitioning into a *C_T_* state under the influence of DAMPs. High values for both means that the initial impact and the subsequent release of DAMPs will have a stronger effect on the dynamics and lead to higher densities of non- *C_U_* cells and higher concentrations of chemicals. The opposite is true for lower values. Low *λ_F_* allows higher saturation of PIC, which leads to more transition of healthy cells into sick cells, as well as higher degradation of the ECM. Therefore, it is not surprising that *S_A_*, *S_T_*, ROS, and DAMPs all significantly increase when *λ_F_* is 0.1. Low *λ_M_* allows for higher saturation of DAMPs. Considering that DAMPs released by the necrotic cells after the impact triggers the whole system, the significant effect of *λ_M_* perturbations on the system is expected. Some differences that resulted from low *λ_M_* are the near eradication of *C_U_* cells and a more pronounced diffusion of the chemicals by day 14.

*S_A_* was the variable most sensitive to parameter perturbations. Considering that they are the last catabolic stage, this is not surprising – any parameter values that increase the transition of *C_U_* to *C_T_* and *C_T_* to *S_T_* would have an indirect effect on the density of *S_A_* cells. Considering that EPO is also, in our model, released as a response to high levels of PIC, by 14 days the concentration of this anti-inflammatory cytokine was not enough to affect the already high level of *S_A_* cells.

## 4 Discussion

We presented a multiscale mathematical model of the balancing act between the pro- and anti- inflammatory cytokines released by the chondrocytes in articular cartilage under a blunt impact from an indenter onto a cylindrical cartilage explant. The complex interplay between cellular behavior and chemical release has been experimentally validated previously (Wang et al., 2015). Briefly, cartilage explants were subjected to a drop tower impact imparting an energy of 2.18 J/cm^2^ and probed immunohistochemically for the presence of interleukin 6 (IL-6, a PIC), erythropoietin (EPO), and the erythropoietin receptor (EPOR) after 1, 7, or 14 days of tissue culture. The results in Wang et al. (2015) qualitatively agreed with the model predictions showing the spatial and time-dependent relationships of the IL-6 and EPO production.

Our current model differs from previous work by incorporating a mechanical finite element simulation and analysis to estimate the role of the blunt impact on the biochemical components of the model. This addition also requires an extra spatial dimension for the cartilage (depth). The model includes three components: chemical, cellular, and mechanical (tissue-scale), instead of the two components of the previous work. The mechanical component describes the strain and cell death resulting from the initial impact. The cellular component describes the different states of the chondrocytes (healthy, sick, dead), and the different chemicals being in these states makes them release. The chemical component represents the interplay between chemicals, their signaling to cells to switch states, and their release by the chondrocytes in a particular state. The mechanical component was modeled using linear elasticity and solved with the finite element solver Abaqus™. The chemical component was modeled by reaction-diffusion equations, and the cellular component was modeled by age-structure, to capture the cellular age of state-switching.

The addition of FE modeling and the extra spatial variable are necessary for creating a model that can be validated by experiments and that can make the jump from explant validation to patient-related predictions. The current set up for FE modeling creates an extra scale for facilitating the addition of biomechanics into a biochemical model. The additional features are non-trivial and required major re-writes of the model software compared to Wang et al. (2015). The additional features led to our results better capturing the cellular behavior from the laboratory experiment from Wang et al. (2015). While the current set-up does not capture all complexities of cartilage biomechanics, it is a significant first step to a more comprehensive modeling framework for creating a virtual piece of cartilage.

**Figure 11:**
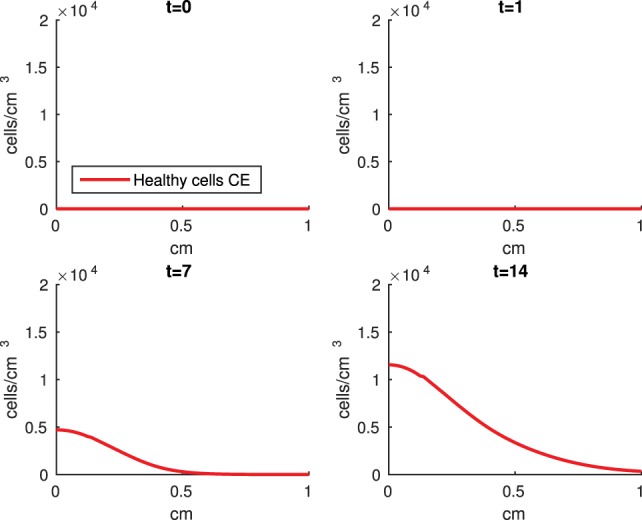
**Density of the erythropoietin producing cells (*C_E_*) at the top layer of the cartilage explant at 0, 1, 7, and 14 days**.

**Figure 12:**
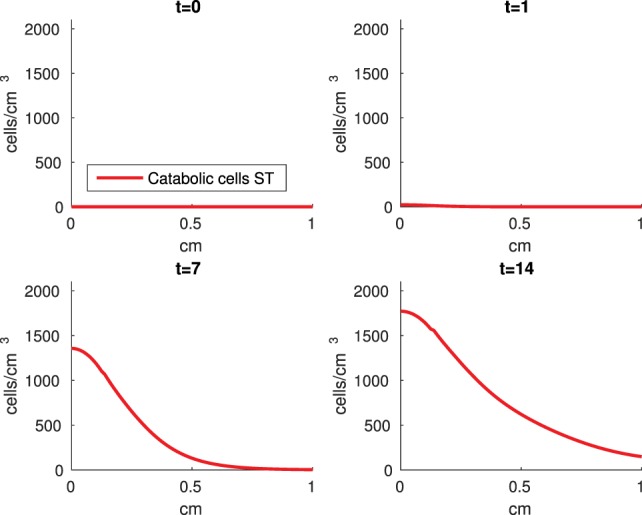
**Density of the pro-inflammatory cytokines producing cells (*S_T_*) at the top layer of the cartilage explant at 0, 1, 7, and 14 days**.

The numerical results of the model simulated the anticipated from the experimental data chemical and cellular behaviors qualitatively well. Concentrations of chemicals and different cellular states are highest around the center of the cylinder, which was expected, given that this is the position of the impact. Therefore, the model is successful in qualitatively estimating the effect of injurious blunt impact application on the cartilage explant for 14 days. The higher density of PIC expressing cells (*S_T_*) in the center of the disk (closer to the injury) and its decrease toward the edges, as well as the overall increase with time, are seen in the *in silico* simulations in Figure 12. This behavior is seen in the experimental validation in Figure 10 in Wang et al. (2015). The results regarding EPO are similar – the higher density toward the center of the cartilage disk and the decrease toward the edges is captured as well [Figure 11, validated by the experimental results shown in Figure 11 in Wang et al. (2015)]. Our model does not capture, however, the densities at day 1. In the experiments, the densities are detectable, while in our model they remain close to zero. A probable explanation is that our initial conditions do not assume levels of EPO and PIC already being produced in the cartilage. For better quantitative estimates we would need a better parametrization, a better understanding of the sensitivity of the system to perturbations of the unknown parameters and the initial strains, and experimental validation. Longitudinal data over a longer time frame would further allow us to validate and calibrate our model to capture the development of OA in the long run. Experiments where the chemical/cellular levels before and after impact are compared will provide us with accurate initial conditions and further calibration of our parameters.

Comparing the results of the 2D model presented here versus the 1D model in Wang et al. (2015), the advantage of including the strain field becomes obvious. In Wang et al. (2015), it is assumed that the blunt impact causes necrosis to all cells within the radius of the indenter. This assumption eliminates the cellular and chemical dynamics at the center of the explant, which in the 2D model appear to be most interesting, and are in fact qualitatively validated by the laboratory experiments, whose results demonstrate that the biochemical reactions related to OA development are exhibited closer to the impact site [Figures 10 and 11 in Wang et al. (2015)]. Therefore, it is important to consider a 2D model of the cartilage explant, and the inclusion of the strain distributions makes the model resemble the experimental results better.

The sensitivity analysis that was conducted does not differ in any major ways from the analysis done in our previous work (Wang et al., 2015). There are two reasons for that. First, we needed to make sure that the addition of an extra dimension did not necessitate vast changes in any parameter magnitudes, considering the complexity of our system. Second, the system is too computationally expensive for a more comprehensive analysis to be conducted at this stage. A lot of methods for parameter estimations and sensitivity are created for and used on systems of ordinary differential equations, which are generally a lot simpler in structure and less resource consuming. Still, we are looking at other ways to conduct our sensitivity analysis in the future. The parameter perturbations provided few surprises in the model’s outcomes. The model’s responses to perturbations were biologically reasonable and expected given the equations and their dependence on the perturbed parameters. Furthermore, the responses were consistent with the one-dimensional model in Wang et al. (2015). Therefore, the addition of the extra spatial dimension does not currently require major modification of the parameter values. Several parameter changes produced different, albeit expected, results: *β*_11_, *β*_13_, *λ*_F_, and *λ*_M_. Considering that most of them affect the strength of the effect of the initial release of DAMPs from the blunt impact, the results are not surprising. Neither is the fact that the density of *S_A_* cells was most sensitive to the parameter perturbations. Being the last catabolic state, all transitions eventually lead to it, hence any parameters that facilitate transitioning facilitates increase in the *S_A_* value (and vice versa). None of the parameters lead to a notable decrease in the ECM concentrations. This leads us to two conclusions. First, an impact of an energy magnitude as the one used in the experiment, is not strong enough to facilitate PTOA within 14 days. This is expected, as even severe intra-articular fractures can take years before radiographic OA is present (Anderson et al., 2011b). Second, that longer timescales (years) would need to be incorporated to see ECM degradation, which at this time are too computationally intensive.

One limitation of the model is that it does not include cartilage heterogeneity. Cartilage explants contain a large portion of subchondral bone, which is not incorporated at this stage. In fact, the actual cartilage layer in an explant is 1–2 mm. More, the development of osteoarthritis has been demonstrated after impact on the subchondral bone, without affecting the cartilage surface (Donohue et al., 1983; Lahm et al., 2004, 2006; Lin et al., 2009). Incorporating the different physical properties of bone and different cartilage layers will alter the stress/strain fields that are used as impact input and, along with the biochemical effect of trauma to the subchondral bone, is the subject of future work. Further, making the model seem more mechanistic by using hydraulics and biphasic elasticity would introduce additional modeling and computational complexity. In the end, we may not have a significantly more accurate model. Furthermore, linear elasticity seems to be a sufficient approximation for the behavior of cartilage under many physiological loading conditions (Carter and Beaupré, 1999). A possible inclusion of more complicated mechanical properties is also a topic for future work. Another limitation to introducing a more complex mechanics is the computational error component. As you can see in Figure 2, the highest displacement is under the surface, when we would expect it to be at the surface of the explant (Johnson, 1985). We believe this phenomenon is due to computational error from the type of induced stress, instantaneous impact. Previous iterations with short term (but not instantaneous) pressure showed highest displacement on the surface of the explant. Further investigation into the methods of our future choice for FEA is warranted. Finally, the relationship between axial strain and cell viability in the current model is simplistic and is taken from a study that evaluates viability after a prolonged period of pressure, rather than a blunt impact (Brouillette et al., 2013). More complex models of the viability of cartilage cells under blunt impact are available, as discussed and presented in Argatov and Mishuris (2015). Generally, a model like the one in Argatov and Mishuris (2015) requires several constants to be fitted to available data. Uniform data of both biomechanical measures (strain) and biochemical measures within our group is currently unavailable and will be a further step in the calibration and validation of the model.

Another current limitation of the model is the fact that several of its parameters are estimated from previous modeling results, and the others are calculated from literature. A goal of ours would be to parametrize the whole model rigorously, using several iterations. First, we would go through accessible literature and revise the previously established parameters. Second, we would attempt to find values from literature for the estimated ones as well. Third, we would use laboratory experiments for calculating parameters. Fourth, we would replicate the experiments in order to create an established list of parameter values related to the project. This process may take years of collaborative work but is a path toward improving the accuracy and predictive power of our modeling efforts. More experiments for calibration and validation of our model are a topic for future work.

The main goal of the presented model was the integration of explicit mechanics, using finite element analysis, into a biomathematical model. The interaction between the finite element simulations and the mathematical modeling of biochemical processes presents an important step toward a comprehensive framework for collaboration between biomedical engineering and mathematical modeling and simulation. This future collaboration will establish biomathematics as an important translational tool from patient-based data to treatment of PTOA. This will involve several steps: using CT scans of join trauma to estimate the mechanical strain of the impact, inputting the strain distribution over the cartilage surface into a finite element solver, which informs a model of the mechanotransductive processes within cartilage. Predicting the progression of PTOA through these steps will require a multiscale modeling platform of enormous magnitude and is the eventual goal of this project. However, developing this platform will require further iterations of the modeling framework and years of calibration and validation of the model parameters, which we intend to do in the future.

## Author Contributions

GK carried out the simulations and analysis, modified the simulation code, and drafted the manuscript. XW helped with developing the mathematical model and modifying the simulation code. BA developed and modified the simulation code, developed the mathematical model, helped to draft and edit the manuscript, and oversaw the project. MB conceived the experimental set-up the simulation is based upon and helped with drafting the manuscript. JM helped with developing the mathematical model and editing the manuscript and oversaw the project. All authors read and approved the final manuscript.

## Conflict of Interest Statement

The authors declare that the research was conducted in the absence of any commercial or financial relationships that could be construed as a potential conflict of interest.

## Abbreviations

DAMPs, damage-associated molecular patterns; ECM, extracellular matrix; EPO, erythropoietin; EPOR, EPO receptor; FE, finite element; OA, osteoarthritis; PIC, pro-inflammatory cytokines; PTOA, post-traumatic osteoarthritis; ROS, reactive oxygen species.
