# Supplementary material for: Linking Cellular and Mechanical Processes in Articular Cartilage Lesion Formation: A Mathematical Model
Source: Front Bioeng Biotechnol. 2016 Oct 31;4:80. doi: 10.3389/fbioe.2016.00080 (PMC5086581; doi:10.3389/fbioe.2016.00080)
Supplement: Supplementary file 1 [file presentation_1.pdf]

Supplementary Material: Linking Cellular and Mechanical Processes in Articular Cartilage Lesion Formation: A Mathematical Model

Georgi I. Kapitanov*, Xiayi Wang, Bruce P. Ayati, Marc J. Brouillette, James A. Martin

*Correspondence:
Author Name: Georgi I. Kapitanov
georgi-kapitanov@uiowa.edu

1 SUPPLEMENTARY TABLES AND FIGURES

1.1 Figures

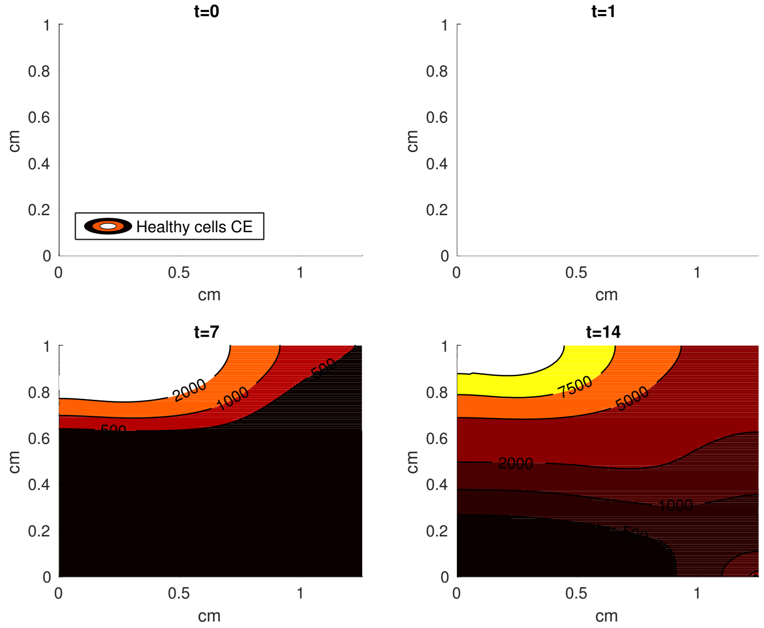

Figure S1. A contour graph of the density of the C_E cells in the 2D model at 0, 1, 7, and 14 days. The units are cells/cm^3.

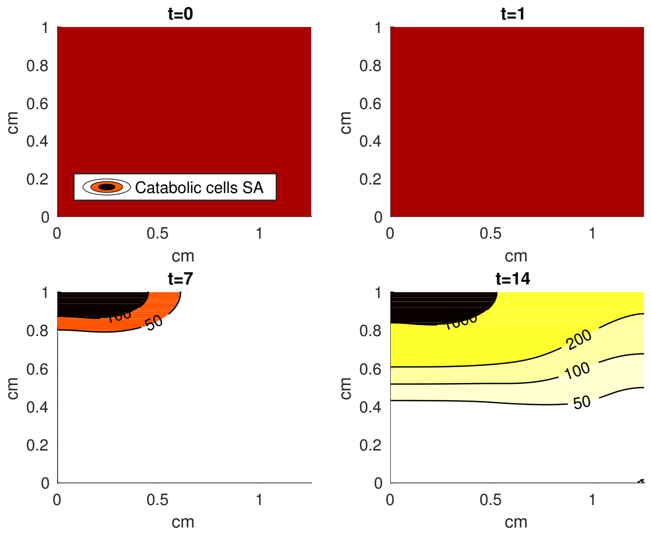

Figure S2. A contour graph of the density of the S_A cells in the 2D model at 0, 1, 7, and 14 days. The units are cells/cm^3.

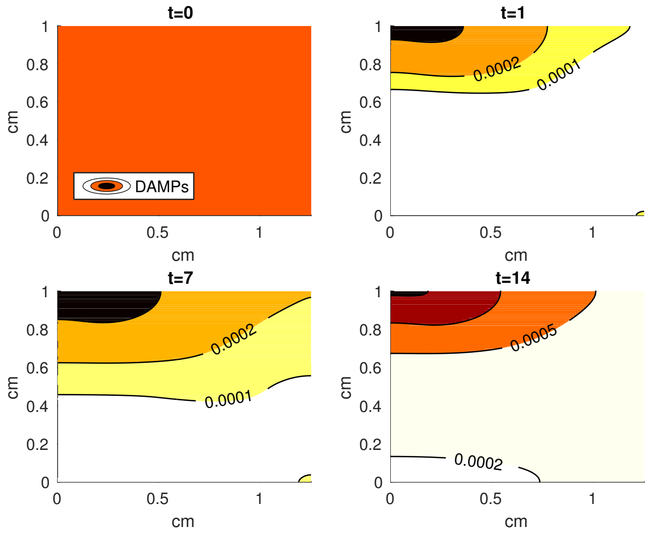

Figure S3. A contour graph of the concentration of DAMPs in the 2D model at 0, 1, 7, and 14 days. The units are nanomolar.

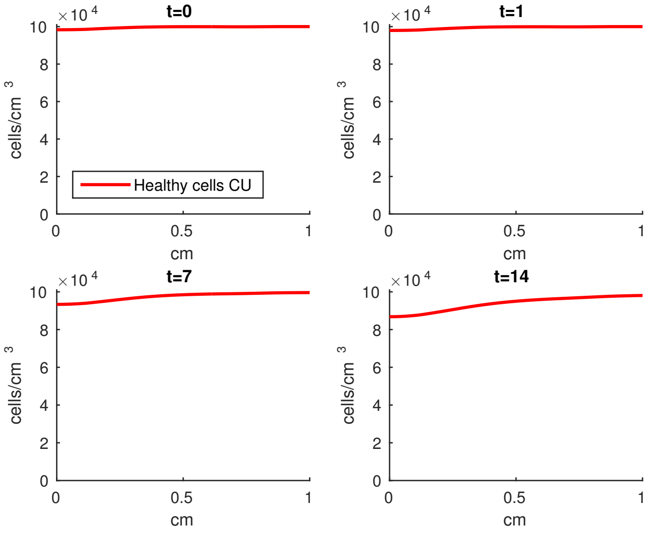

Figure S4. Average density of C_U cells along the radius of the model cylinder at 0, 1, 7, and 14 days. The C_U cells are less dense near the area of contact.

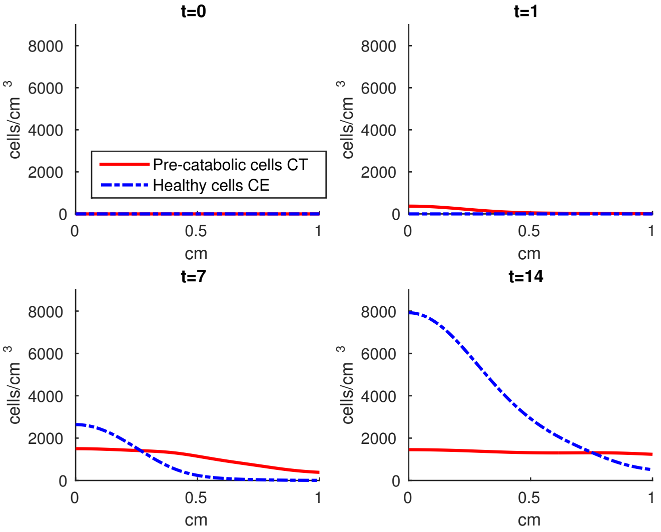

Figure S5. Average density of C_T and C_E cells along the radius of the model cylinder at 0, 1, 7, and 14 days. The density of C_E cells is higher near the contact area, while the density of the C_T cells is more evenly distributed.

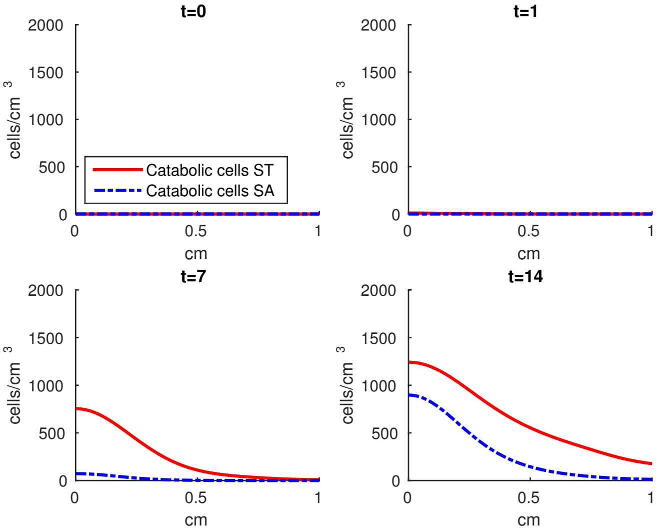

Figure S6. Average density of S_T and S_A cells along the radius of the model cylinder at 0, 1, 7, and 14 days. The densities of both cell states are higher near the contact area.

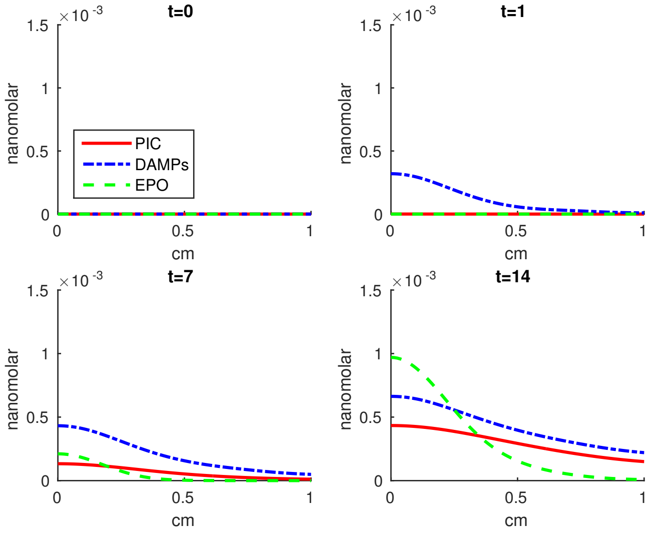

Figure S7. Average concentrations of EPO, DAMPs, and PIC along the radius of the model cylinder at 0, 1, 7, and 14 days. DAMPs and particularly EPO concentrations are higher near the contact area. PIC concentrations are more evenly spread, although their concentrations are also somewhat higher near the area of contact.

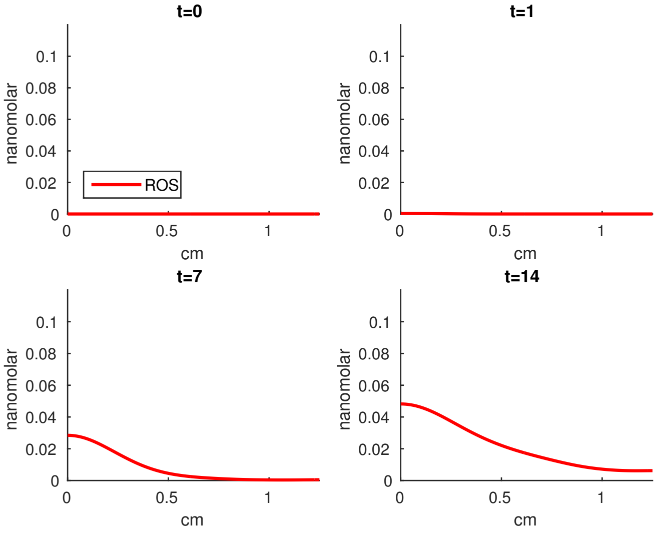

Figure S8. Average concentrations of ROS along the radius of the model cylinder at 0, 1, 7, and 14 days. The concentrations are higher near the area of contact.

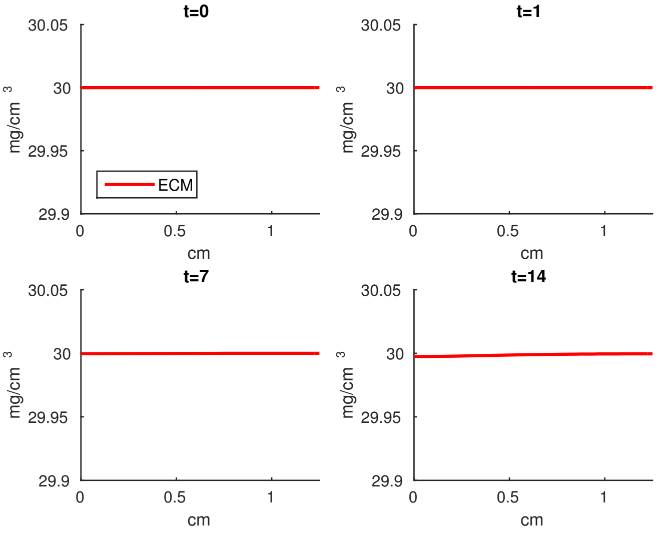

Figure S9. Average ECM density along the radius of the model cylinder at 0, 1, 7, and 14 days. The pressure applied is not enough to produce high deterioration of the ECM.
